# 960. Clinical impact of follow-up blood cultures in gram-negative bloodstream infections: A validation cohort

**DOI:** 10.1093/ofid/ofad500.021

**Published:** 2023-11-27

**Authors:** Joshua T Thaden, Felicia Ruffin, Joshua B Parsons, Vance G Fowler, Stacey Maskarinec

**Affiliations:** Duke University School of Medicine, Durham, North Carolina; Duke University Medical Center, Durham, North Carolina; Duke University Medical Center, Durham, North Carolina; Duke University Medical Center, Durham, North Carolina; Duke University Medical Center, Durham, North Carolina

## Abstract

**Background:**

Obtaining follow-up blood cultures (FUBCs) to document clearance of gram-negative bloodstream infections (GN-BSI) is controversial. In a historical cohort (2002-2015), we previously showed that obtaining FUBC was associated with decreased mortality. However, it is unclear if this result is applicable to present-day patients given changes in epidemiology, practice patterns, and antibiotic susceptibility. Here we used a modern cohort of patients (2015-2020) to determine if obtaining FUBC is associated with patient mortality.

**Methods:**

All unique, adult inpatients with GN-BSI at Duke from 2015-2020 were prospectively enrolled. FUBC was defined as those obtained from 24h to 7d after initial positive blood culture. Patients that died < 24h after initial positive culture were excluded. Outcomes were compared with Fisher’s exact tests. Multivariable Cox proportional hazards models were used to compare outcomes with adjustment for clinical variables. To account for immortal time bias, FUBCs were treated as a time-dependent variable.

**Results:**

Of the 870 patients in this study, FUBCs were obtained in 672 (77%). Obtaining FUBCs was associated with decreased total in-hospital mortality (140/672 [21%] vs 64/198 [32%]; p=0.001) and attributable (i.e., death due to BSI) mortality (48/672 [7%] vs 32/198 [16%]; p=0.0003). Patients in whom FUBCs were and were not obtained differed with respect to age, history of transplant, hospital acquisition of BSI, and source of BSI (Table 1). After adjustment for patient clinical variables, obtaining FUBC remained associated with decreased patient in-hospital mortality (OR 0.67, 95% CI 0.47-0.95; p=0.02; Fig 1). Among 672 patients in whom FUBCs were obtained, 121 (18%) had positive FUBCs with the same bacterial species. In patients with FUBCs, a positive FUBC was associated with increased total in-hospital (35/121 [29%] vs 105/551 [19%]; p=0.02) and attributable mortality (14/121 [12%] vs 34/551 [6%]; p=0.05).

Table 1

**Figure:**
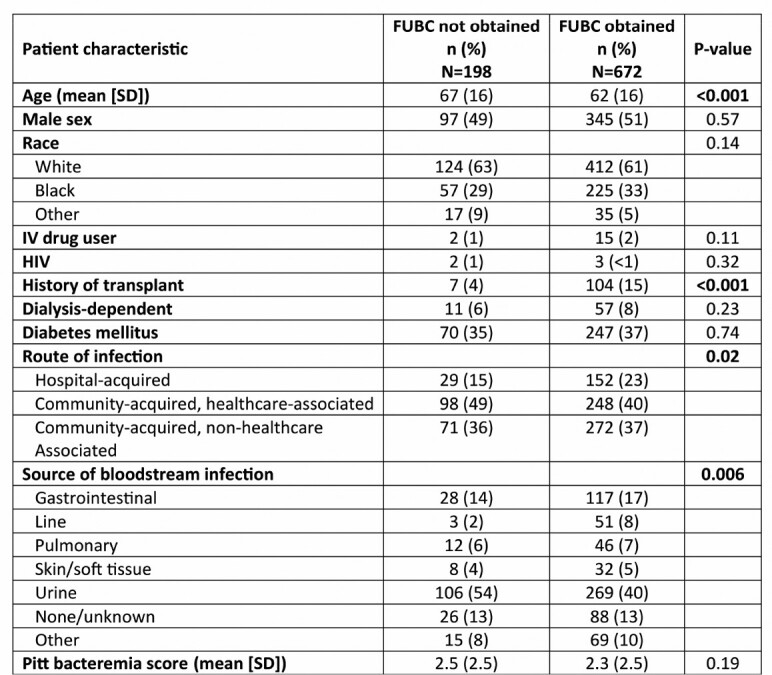

Characteristics of patients in whom follow-up blood cultures (FUBCs) were and were not obtained.

Figure 1

**Figure:**
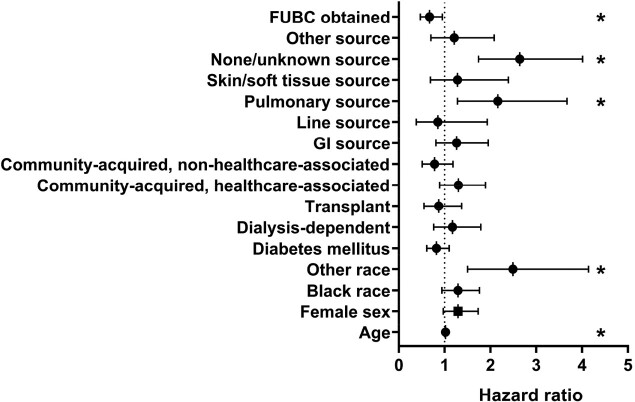

Cox proportional hazards model total in-hospital mortality in patients with gram-negative bloodstream infections. The hazard ratio and 95% confidence intervals are presented. Variables associated with mortality (p<0.05) are marked with an asterisk (*). A hazard ratio greater than 1 indicates increased mortality, while a hazard ratio less than 1 indicates decreased mortality. Obtaining follow-up blood cultures (FUBC) was associated with decreased mortality. The reference for source of infection is urinary tract. The reference for route of infection was hospital-acquired. The reference for race is White race.

**Conclusion:**

In this modern cohort of patients, obtaining FUBC was common and associated with decreased mortality. These results are in line with our previous historical cohort. The mortality difference could stem from identification of patients with positive FUBC, which is a poor prognostic indicator.

**Disclosures:**

**Joshua T. Thaden, MD, PhD**, Resonantia Diagnostics, Inc: Advisor/Consultant **Vance G. Fowler, MD, MHS**, Amphliphi Biosciences, Integrated Biotherapeutics; C3J, Armata, Valanbio; Akagera, Aridis, Roche, Astra Zeneca: Advisor/Consultant|Genentech, Regeneron, Deep Blue, Basilea, Janssen;: Grant/Research Support|Infectious Diseases Society of America: Honoraria|MedImmune, Allergan, Pfizer, Advanced Liquid Logics, Theravance, Novartis, Merck; Medical Biosurfaces; Locus; Affinergy; Contrafect; Karius;: Grant/Research Support|Novartis, Debiopharm, Genentech, Achaogen, Affinium, Medicines Co., MedImmune, Bayer, Basilea, Affinergy, Janssen, Contrafect, Regeneron, Destiny,: Advisor/Consultant|Sepsis diagnostic: Patent pending|UpToDate: Royalties|Valanbio and ArcBio: Stock Options **Stacey Maskarinec, MD, PHD**, National Institutes of Health: Grant/Research Support

